# Age as a limiting factor for effectiveness of photostimulation of brain drainage and cognitive functions

**DOI:** 10.1007/s12200-025-00149-3

**Published:** 2025-03-31

**Authors:** Terskov Andrey, Shirokov Alexander, Blokhina Inna, Zlatogorskaya Daria, Adushkina Viktoria, Semiachkina-Glushkovskaia Anastasiia, Atul Kumar, Fedosov Ivan, Evsukova Arina, Semyachkina-Glushkovskaya Oxana

**Affiliations:** 1 Department of Biology, Saratov State University, Saratov, 410012 Russia; 2 Institute of Biochemistry and Physiology of Plants and Microorganisms, Russian Academy of Sciences, Saratov, 410049 Russia; 3 Department of Computer Science and Information Technology, Saratov State University, Saratov, 410012 Russia; 4 The Indian Institute of Technology (BHU) Varanasi, Uttar Pradesh, Varanasi, 221005 India; 5 Institute of Physics, Saratov State University, Saratov, 410012 Russia

**Keywords:** Photobiomodulation, Aging brain, Meningeal lymphatic vessels, Brain drainage, Cognitive functions

## Abstract

The progressive number of old adults with cognitive impairment worldwide and the lack of effective pharmacologic therapies require the development of non-pharmacologic strategies. The photobiomodulation (PBM) is a promising method in prevention of early or mild age-related cognitive impairments. However, it remains unclear the efficacy of PBM for old patients with significant age-related cognitive dysfunction. In our study on male mice, we show a gradual increase in the brain amyloid beta (Aβ) levels and a decrease in brain drainage with age, which, however, is associated with a decline in cognitive function only in old (24 months of age) mice but not in middle-aged (12 months of age) and young (3 month of age) animals. These age-related features are accompanied by the development of hyperplasia of the meningeal lymphatic vessels (MLVs) in old mice underlying the decrease in brain drainage. PBM improves cognitive training exercises and Aβ clearance only in young and middle-aged mice, while old animals are not sensitive to PBM. These results clearly demonstrate that the PBM effects on cognitive function are correlated with age-mediated changes in the MLV network and may be effective if the MLV function is preserved. These findings expand fundamental knowledge about age differences in the effectiveness of PBM for improvement of cognitive functions and Aβ clearance as well as about the lymphatic mechanisms responsible for age decline in sensitivity to the therapeutic PBM effects.

**Graphical Abstract:**

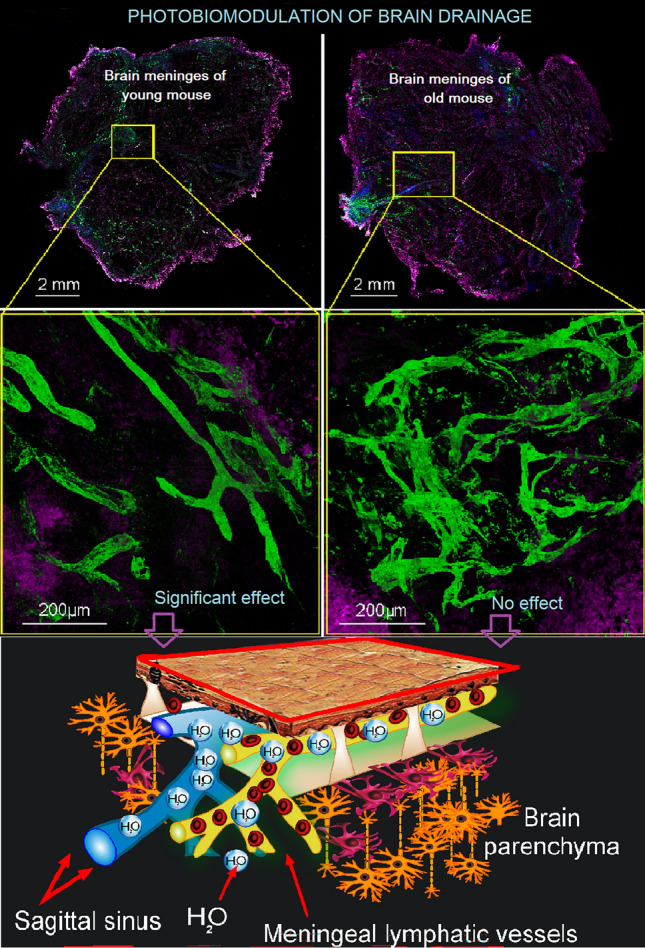

## Introduction

Age is a biological factor triggering irreversible changes in brain tissue and functions [1–3]. The most common age-related manifestations are memory and cognitive decline [4, 5]. Age-related changes in brain functions are a long-term process and in the early and even middle stages can be improved by transcranial photobiomodulation (PBM) [6–12]. However, despite promising results in PBM-mediated cognitive improvement in middle-aged and elderly people, when only the first signs of cognitive dysfunction are evident, the efficacy of PBM in old people, when cognitive impairment and brain aging are progressive, remains poorly studied.

Meningeal lymphatic vessels (MLVs) are one of the targets for PBM [13–18]. MLVs have an important function in regulation of brain drainage and the excretion of metabolites and toxins, such as amyloid beta (Aβ), through the fluid stream [19]. A number of studies have found that the function of MLVs decreases with age, which may lead to Aβ accumulation in brain tissue as well as cognitive decline [18–20]. Indeed, aged mice demonstrated significant regression in branches of dorsal MLVs leading to suppression of drainage of the cerebral spinal fluid [20]. Aging results in morphological (decreased lymphatic vessel density) and functional (reduced transport capacity) alterations in the lymphatic vessels including thoracic duct, skin, meningeal and mesenteric lymphatic network [19–21]. The main reason of age-related alteration of the lymphatic vessels is lower production of lymphangiogenic factors and diminished regenerative capacity of the lymphatic endothelium [19, 20]. Aging also reduces the lymphatic smooth muscle actin coverage around valves leading to decreasing contractility of the lymphatic vessels [22–26]. There is evidence that the reduced MLV function is an important factor associated with the development of Alzheimer’s disease [16, 19, 27]. So, transgenic 5xFAD mice demonstrate diminished meningeal lymphatic coverage of the main venous sinuses that are accompanied with a significant Aβ deposits in brain tissue and cognitive decline [19]. The damage of MLV results in significant Aβ accumulation in the brain [15, 16, 19]. Thus, age-related changes in MLVs can impair brain drainage leading to accumulation of metabolites and toxins.

PBM has stimulatory effects on the MLV functions, improving lymphatic excretion of various toxins from the brain, such as Aβ in mice with Alzheimer’s disease [15, 16, 18, 28] and blood products in mice with a model of intraventricular hemorrhage [13].

PBM helps to increase the contractility of the lymphatic vessels thereby improving the lymph flow [13]. Two factors, such as nitric oxide (NO) and calcium (Ca^2+^)-channels, regulate peristaltic contractions of the lymphatics vessels and the functions of the lymphatic valves, which ensures unidirectional lymph flow [29–32]. There is a hypothesis based on experimental data that PBM-effect on the endothelium of basal MLVs leads to the generation of ^1^O_2_ in the mitochondria, which is accompanied by the NO formation, mainly in the valves because 50% of the endothelial NO-synthase is localized there [29, 33–35]. The described effects can be related to a PBM-mediated increase in the activity of endothelial synthase (eNOS) [36]. The NO is a vasodilator that acts via stimulation of soluble guanylate cyclase to form cyclic-GMP, which activates protein kinase G causing the opening of calcium-activated potassium channels and reuptake of Ca^2+^. The decrease in the concentration of Ca^2+^ prevents myosin light-chain kinase from phosphorylating the myosin molecule, leading to the relaxation of lymphatic vessels [37].

Traditionally, NO is considered to only relax the vascular endothelium. However, it is also a factor that triggers vascular contractility [29–32]. Liao et al. revealed that eNOS in lymphatic endothelial cells is required for robust lymphatic contractions under physiologic conditions [38]. PBM-mediated release of NO stimulates dilation of MLVs and increases their permeability, which leads to an increase in their volume due to the influx of fluid into them [13]. In this moment, the upstream valve is open, and the downstream valve is closed. When MLVs are filled, share stress decreases and NO is degraded. Afterward, a subsequent contraction of MLVs is initiated through Ca^2+^ influx both via stretch-, voltage-, or ion-activated channels and from the depot. The contraction of MLVs closes the upstream valves and opens the downstream valves leading to an increase in a wall shear stress and the NO production locally, thus starting the cycle again. This way is the peristaltic process in MLVs, which is the basis of their drainage and cleansing functions [33].

In recent studies on young mice, improved brain drainage and the MLV functions after a course of PBM has been shown to promote better learning [16, 17]. PBM is also effective in reducing the brain Aβ levels in adult healthy mice and animals with Alzheimer’s disease [15, 16, 18].

However, with age, there are significant changes in the morphology of MLVs leading to a dramatic decline in their functions [19, 20]. It is believed that with age, the lymphatic valves lose the ability to effectively control peristaltic contractions of the lymphatic vessels leading to significantly reduction of lymph flow [39–42].

Because most studies of PBM-mediated cognitive improvement have been performed on adult or middle age subjects [6–12], it remains unclear whether PBM is effective in late ontogenesis when there is significant progression of cognitive impairment. For example, there were no therapeutic effects in some old patients (85 years old) with idiopathic Parkinson’s disease and with mild- and moderate Alzheimer’s disease after PBM during 12 weeks [43, 44]. To answer this question, we investigated the effects of PBM (daily 10-day course, 1050 nm, 30 J/cm^2^ in pulsed mode) on cognitive training exercises in 3-12-24-month-old mice corresponding to 25-42.5-69 human ages [45, 46]. To understand the mechanisms of age-related differences in PBM sensitivity, age-related changes in the MLV network, brain drainage and the Aβ brain levels were investigated.

## Methods

### Subjects

Male C57BL/6 mice (3-12-24-month-old, 25-28-35 g, respectively) were used in all experiments and were obtained from the National Laboratory Animal Resource Centre in Pushchino (Moscow area, Russia). The choice of mouse age is related to the evaluation of PBM efficacy in young (3 months old), middle-aged (12 months old) and in old (24 months old) mice, corresponding to 25, 42.5 and 69 years of age in humans [45, 46]. The animals were housed under standard laboratory conditions with access to food and water ad libitum. All experimental procedures were performed in accordance with the “Guide for the Care and Use of Laboratory Animals,” Directive 2010/63/EU on the Protection of Animals Used for Scientific Purposes, and the guidelines from the Ministry of Science and High Education of the Russian Federation (Nº 742 from 13.11.1984), which have been approved by the Bioethics Commission of the Saratov State University (Protocol No. 8, 18.04.2023). The mice were housed at (25 ± 2)°C, 55% humidity, and 12:12 h light–dark cycle (light: 20:00–08:00). The mice adapted to the experimental conditions during one week before the beginning of the experiments to ensure acclimation to the housing room of the animal facility. The study of cognitive function and the Aβ brain levels was conducted in the following groups: (1–3) intact 3-12-24 month-old mice, respectively; (4–6) 3-12-24-month-old mice receiving the 10-day PBM course, respectively. The study of mechanisms of age-related differences in sensitivity to PBM, including the optical monitoring of tracer distribution in the brain and its lymphatic release into the deep cervical lymph nodes (dcLNs), as well as assessment of the MLV network, was carried out on intact mice of different ages (3-12-24 months). *n* = 8–10 in each group in all sessions of the experiments; the total number of animals used in the study was 114.

### Immunohistochemical assay and confocal imaging of the MLV network

For confocal imaging of the meninges, we used the protocol for the immunohistochemistry analysis with the lymphatic vessel endothelial hyaluronan receptor 1 (LYVE1), which a marker of the lymphatic endothelium. The meninges were collected in accordance with the previously published protocol [47, 48]. The meninges were fixed for 48 h in a 4% saline solution-buffered formalin. The antigen expression was evaluated on the meninges according to the standard method of simultaneous combined staining (abcam protocols). The nonspecific activity was blocked by 2 h incubation at room temperature with 10% BSA in a solution of 0.2% Triton X-100 in the phosphate-buffered saline (PBS). Incubation with primary antibodies in a 1:500 dilution took place overnight at 4°C with rabbit anti-Lyve-1 antibody (1:500; ab 218535, Abcam, Cambridge, UK). At all stages, the samples were washed 3–4 times with 5-min incubation in a washing solution. Afterward, the corresponding secondary antibodies goat anti—rabbit IgG (H + L) Alexa Flour 488 (Invitrogen, Molecular Samples, Eugene, Oregon, USA) were applied. At the final stage, the meninges were transferred to the glass and 15 µL of mounting liquid (50% glycerin in PBS) was applied. In the final step, the meninges were covered with a cover glass and confocal microscopy was performed.

The meninges were visualized using a confocal microscope LEICA TCS SP8 (Leica Microsystems, Wetzlar, Germany) with a × 20 lens (0.75 NA) or a × 100 lens for immersion in oil (0.45 NA). DAPI, Alexa Fluor 488 were excited with excitation wavelengths of 405 nm, 488 nm, respectively. Three-dimensional visualization data were collected by software 3.0.16120.2 LAS X (Leica Microsystems, Germany) and analyzed using Fiji software 2.0.0 (Open-source image processing software) [49].

### Ex vivo study of brain drainage

To study the PBM effects on brain drainage in mice of different ages, dye spreading in the dorsal and ventral parts of brain after a single PBM application was analyzed. An amount of 5 μL of fluorescein isothiocyanate (FITC)-dextran (FITCD 70 kDa, Sigma, St Louis, United States) was injected into the right lateral ventricle (AP-1.0 mm; ML-1.4 mm; DV-3.5 mm) at a rate of 0.1 μL/min using microinjector (Stoelting, St. Luis, USA) with a Hamilton syringe with a 29-G needle (Hamilton Bonaduz AG, Switzerland). The implantation of chronical polyethylene catheter (PE-10, 0.28 mm ID × 0.61 mm OD, Scientific Commodities Inc., Lake Havasu City, Arizona, USA) into the right lateral ventricle was preformed according to the protocol reported by Devos and Miller [50]. The ex vivo optical study of FITCD distribution in the brain fluid system was performed 1.5 h after the intracerebroventricular injection of FITCD. Afterward, we performed ex vivo optical imaging of the dorsal/ventral aspects of the brain and dcLNs.

The imaging was performed using Nikon A1R MP upright confocal microscope (Nikon Corp., Tokyo, Japan) with CFI Plan Apochromat Lambda D 2X (Nikon Corp., Tokyo, Japan) installed in a focusing nosepiece. Brain samples were submerged in a buffer solution in a Petri dish placed on the microscope stage. The top surface of each sample was covered with a 25 mm × 25 mm × 0.17 mm glass cover slide. Each brain image was captured as a stack of 5 stitched large images over 4 × 4 fields of view each. Image resolution was 3636 × 3636 pixels at 6.11 µm/pixel. Z step was 222 μm. The resulting image was obtained as maximum intensity Z projection of all 5 images of the stack. The method enables to obtain extended along 1 mm depth of focus of high resolution image. The confocal images were captured in two channels: 488 nm excitation/525 emission was used to image FITCD distribution; and 640 nm excitation/700 nm emission for Evans Blue dye. dcLNs were imaged identically but only single field of view was captured for each stack. Image processing was performed using Fiji open-source image 2.0.0 processing package [49]. Image processing procedures were identical for each pair of images (control and laser-treated samples) for each channel to ensure an accurate comparison of the fluorescence intensity.

### PBM protocol

The head plate with light-emitting diode (LED) was fixed in the region of the parietal and interparietal bones using dental acrylic (Zhermack SpA, Badia Polesine, Italia) under inhalation anesthesia with 1% isoflurane (Sigma-Aldrich, St Luis, USA, at rate 1 L/min N_2_O/O_2_—70/30 ratio). The LED was fixed to the head plate with two screws and was places on the parietal region, where the largest number of MLVs is located [18, 20]. The LED was performed during 61 min using following sequences: 17 min—LED, 5 min—pause, 17 min—LED, 5 min—pause, 17 min—LED as we have determined by a random selection in our previous studies [15, 17, 18]. To study the PBM effects on the brain Aβ levels or cognitive functions, we used the 10-day course of PBM (0.3 kJ/cm^2^ per 10 days, 30 J/cm^2^ for 17 min of PBM exposure, the irradiance at skull surface does not exceed 0.5 W/cm^2^). The 10-day course of PBM was started from the first day of training mice and formation of conditioned reflexes in them as described in paragraph 2.5.

### Pavlovian and instrumental conditioning

**Fig. 1:**
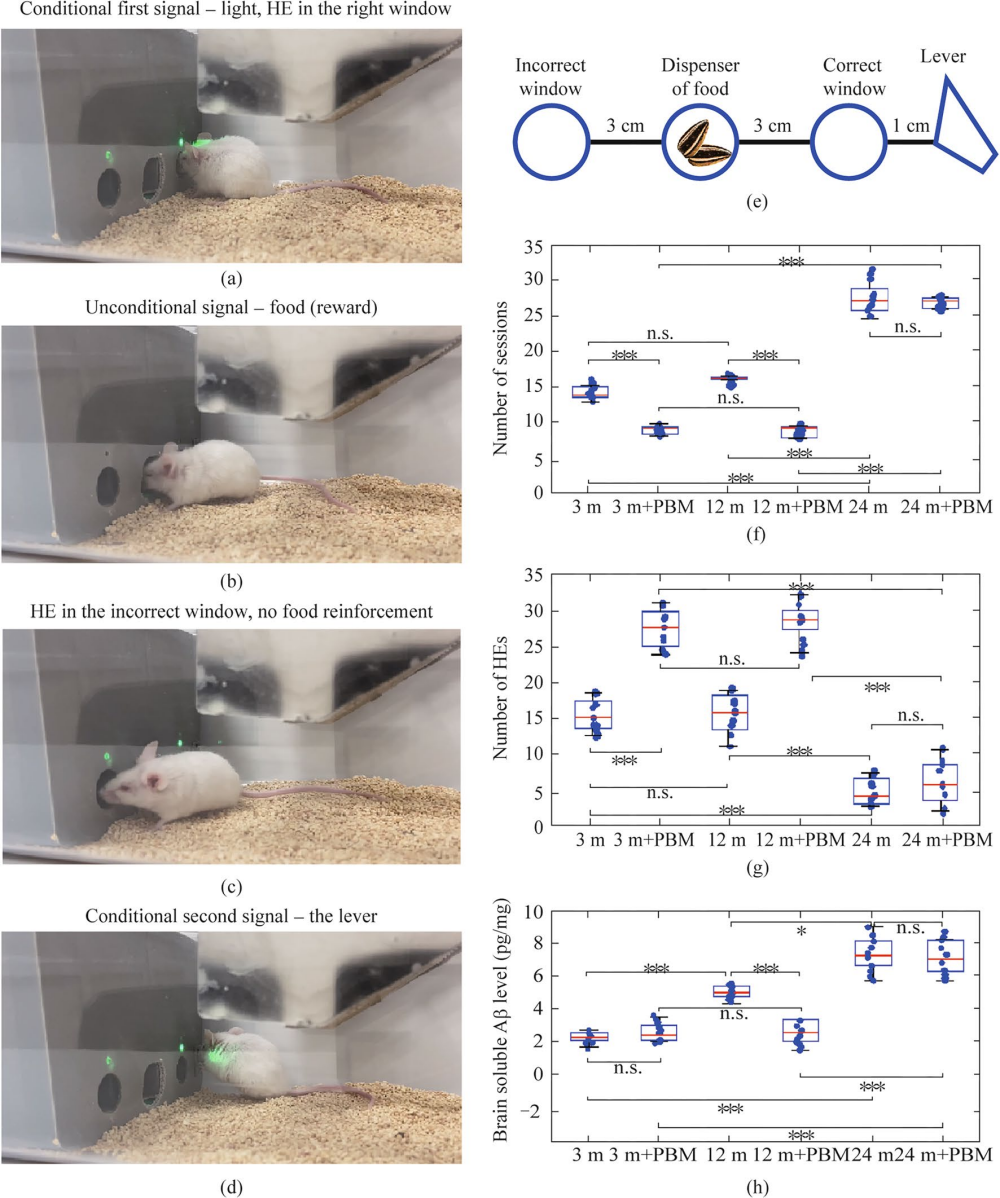
PBM effects on the PCR formation and the soluble Aβ brain levels in mice of different ages. **a**–**d** Illustration of the stages of the PCR formation. **e** Schematic representation of the operative wall used for mouse training and the PCR formation. **f** and **g** Quantitative analysis of the effects of the 10-day course of PBM on learning in the tested groups, including evaluation of session’s numbers (**f**) that were required to reach PCR and the number of HEs (**g**) in the food dispenser (the middle window) reflecting successful reinforcement with food of two conditioned signals “light-lever”; **h** Quantitative analysis of the effects of the 10-day course of PBM on the soluble Aβ brain levels the tested groups; *n* = 10 in each group, the ANOVA test with post hoc Duncan test, *p < 0.05; ***p < 0.001 between groups, ns means not significant

Here we used a protocol, which was adapted and published earlier [17, 51, 52]. Pavlov’s conditioned reflex (PCR) (light-food) was developed according to the principle of reinforcement a conditioned signal (light) by an unconditioned one (food). The test was conducted in the operant chamber (25 cm × 20 cm × 15 cm) housed within light (infrared LED) located inside the receptacle to detect head entries (HEs). There were three windows located at a distance of 3 cm from each other along a horizontal line (Fig. 1a–e). One in the middle—serves to dispense a food (reward), the other two on the left (incorrect) and right (correct) sides—equipped with infrared sensors, which allow to detect HEs. 1 s after HE, a signal from the correct window stimulated to give a reward (food), while HE in the incorrect window did not result in food release (Fig. 1a–c).

A pellet dispenser for delivery of 12-mg reward pellets (seeds of sunflowers, Grums, Minsk, Belarus) was located inside of the receptacle in the center of chamber (Fig. 1b). Green light as a conditioned signal was given above both the correct and incorrect windows. The choice of green light was related to the fact that mice have only two types of cone cells, some of which respond to green light and others to ultraviolet light [53]. The mice were trained every day until stable formation of PCR. The duration of one session was 30 min. A stable PCR was considered when the mouse successfully received food within 15 s after being placed in the operant chamber and given a conditioned signal (light). When PCR (light-food) became stable, training was conducted to transfer the educated experience to new experimental conditions, when after the light the food was not given out after 15 s, but only with a delay of 30 s, so that the mouse learned to press the ultrasensitive lever additionally (Fig. 1d). Thus, the conditioned reflex became more complex “light-lever-food.” The assessment of learning was carried out by the automatic analysis of the number of sessions (days) required for the stable formation of PCR “light-lever-food,” as well as successful reinforcement with food of two conditioned signals “light-lever” estimated as the number of NEs in the food dispenser (the middle window). It should be noted that before the start of training, mice adapted to the operant chamber and to the hands of the experimenter for 2 weeks.

### ELISA analysis of the Aβ brain levels

For ELISA, a kit for the determination of the soluble form of Aβ (1–42) (Cloud Clone, no. CEA946Mu) was used. The brains from all tested groups were homogenized and lysed for further peptide isolation and preparations using Cloud Clone Protocol. In total, lysates from brain tissues were prepared in a lysing buffer (1.5 mm KH_2_PO_4_, 8 mm Na_2_HPO_4_, 3 mm KCl, 137 mm NaCl, and 0.1% Twin20, 10 mM EDTA), pH 7.2, with a freshly prepared protease inhibitory mixture (Roche Applied Science). Measurements of the optical density of the studied samples were carried out at a wavelength of 450 nm (A450) on an automatic enzyme immunoassay analyzer–microplate spectrophotometer Epoch (BioTek Instruments, Winooski, Vermont, USA). The obtained results were subjected to statistical processing. Confidence intervals were determined for 95% of the significance level. The processing of experimental data with a sample size (*n* = 8–10) was carried out by the method of univariate analysis of variance ANOVA.

### Statistical analysis

All statistical analysis was performed using Microsoft Office Excel and SPSS 17.0 for Windows software. The results were reported as a mean value ± standard error of the mean (SEM). The inter-groups differences in all series experiments were evaluated using the ANOVA test with post hoc Duncan test. The significance levels were set at *p* < 0.05 for all analyses.

## Results

### PBM improves cognitive function in young and middle-aged but not in old mice

In the first step, we answered the question whether there was a difference in the effects of PBM on cognitive function in male mice of different ages. The formation of new PCR, which is complex behavior, based on a combination of memory and thinking due to the formation of new synaptic connections [54, 55], was chosen as a test of cognitive abilities. Mice learn to respond to an unfamiliar conditional signal (light), receiving reward (food), and when the “light-food” PCR becomes stable, learning is complicated by the need to transfer experience to new condition, when the mouse receives food after responding to two consecutive conditional signals “light-lever,” due to which a new instrumental PCR “light-lever-food” reflex is formed (Fig. 1a–d).

The results are presented in Fig. 1f and g clearly demonstrate that the formation of PCR did not differ between 6- and 12-month-old mice, while 24-month-old animals performed this test much longer. Indeed, the number of sessions (days) required for both training (PCR “light-food”) and transfer of experience to new environment (PCR “light-lever-food”), as well as the number of rewards (food), was higher in young and middle-aged mice compared to old animals.

Importantly, PBM significantly increased the rate of PCR formation (“light-lever-food”) in 3- and 12-month-old mice, but not in 24-month-old animals (Fig. 1f and g).

Thus, the results of this series of studies suggest that PBM can improve learning in young and middle-aged mice, but is not effective in improvement of cognitive abilities in old mice.

### Age-related changes in the MLV network

Since MLV are the targets of PBT, the next step was to test the hypothesis that age-related changes in MLVs could be the cause of the lack of sensitivity to PBT in old mice.

**Fig. 2:**
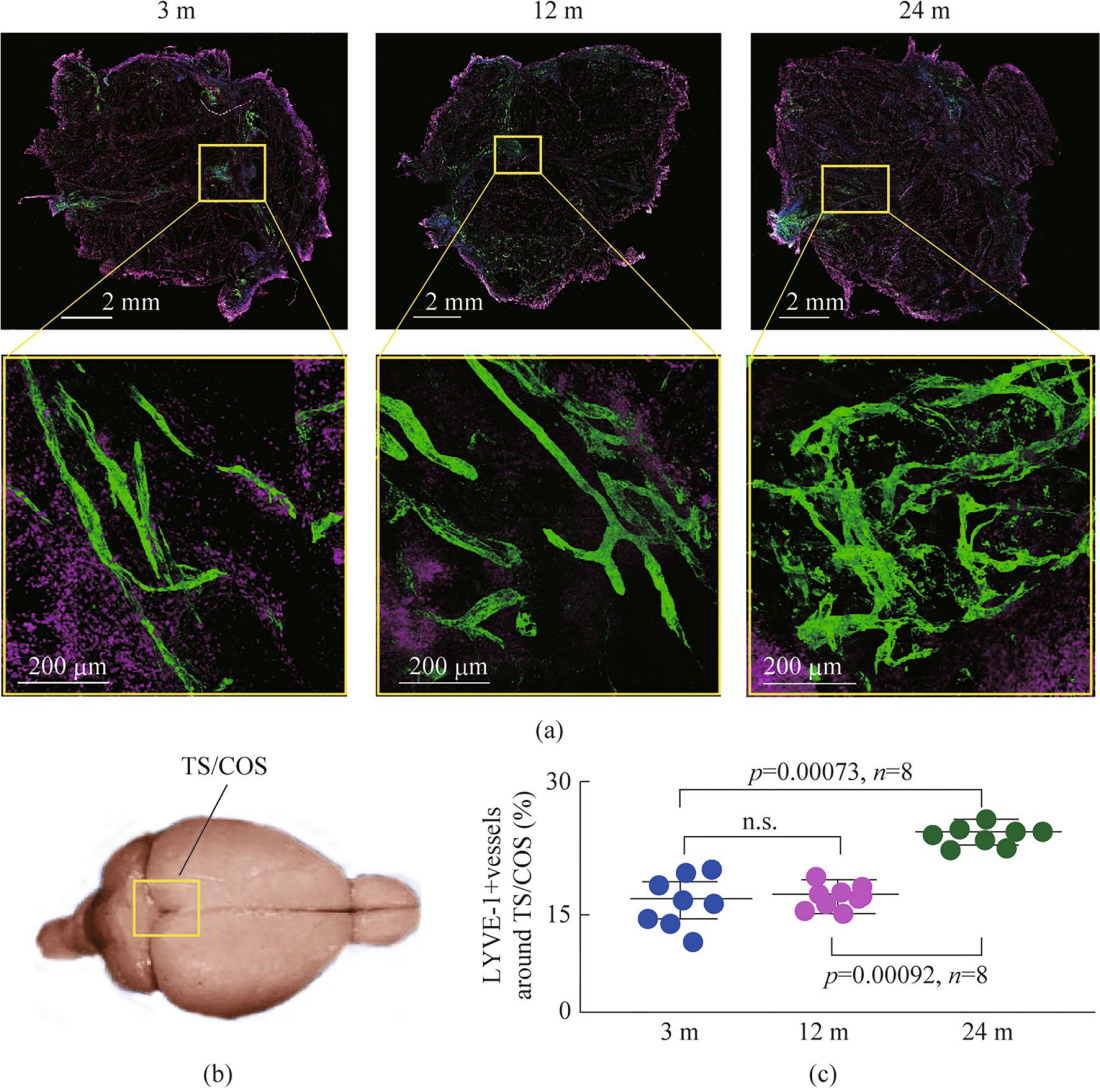
Age differences in the MLV network. **a** Representative images of the LYVE-1 + vessels (green) covering the main venous sinuses (TS/COS) in 3-12-24-month-old mice, DAPI are colored pink. **b** Schematic illustration of ROI for the confocal analysis of MLVs. **c** Quantitative analysis of LYVE-1 coverage expressed in % of ROI, *n* = 8 in each group, the ANOVA test with the post hoc Duncan test

To study age-related changes in the MLV network, we examined the representation of LYVE-1-positive vessels around the main venous sinuses (the transverse (TS) and the confluence of sinuses (COS)) using confocal microscopy (Fig. 2a and b). The results revealed that 24-month-old mice exhibited a higher representation of the LIVE-1 + vessels compared with 3- and 12-month old mice (Fig. 2a and c). There were no significant differences in the density of LYVE-1-positive vessels around the venous sinuses between young and middle-aged mice. Thus, the findings demonstrate significant changes in the MLV network, manifested as lymphatic hyperplasia, in old, but not in young and middle-aged mice.

### Age-related changes in brain drainage and the Aβ brain levels: the PBM effects on Aβ clearance

Age-related changes in the MLV network inevitably affect their function. As noted above, the overgrowth of the lymphatic endothelium in old mice can be associated with a decrease in lymph flow in MLVs [20]. Based on this hypothesis, it is logical to assume a decrease in brain drainage in old mice. Therefore, in the next step, we studied age differences in distribution of FITCD in brain tissues and its lymphatic drainage to dcLNs after an injection of tracer into the cisterna magna.

The MLVs are “tunnels” for the excretion of Aβ, which is a metabolite of neurons and is formed in large amounts daily [56, 57]. Therefore, the brain soluble Aβ content reflects the efficiency of lymphatic regulation of brain drainage. To test the PBM effects on brain drainage, we additionally studied the soluble Aβ levels in the brains of mice of different ages before and after 10-day course of PBM (Fig. 1h).

**Fig. 3:**
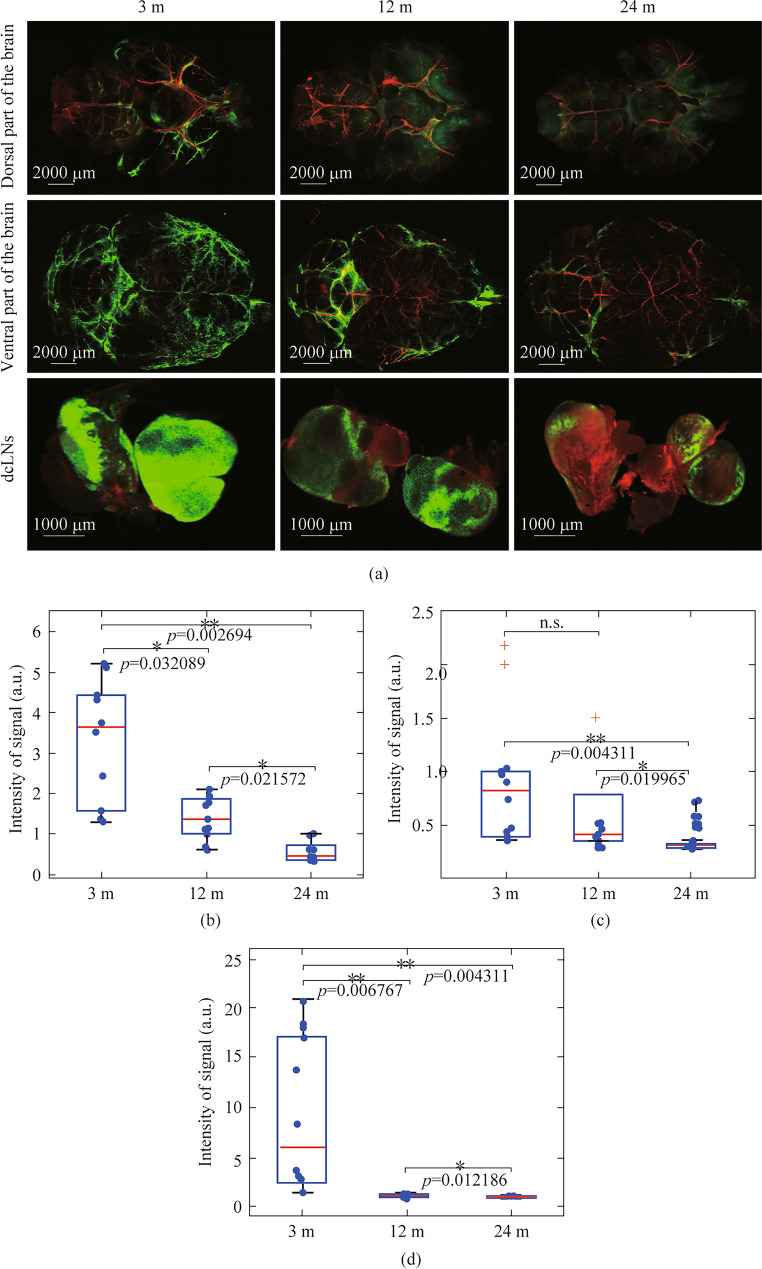
Age differences in brain drainage. **a** Representative images of FITCD (green) distribution in dorsal and ventral parts of the mouse brains as well as accumulation of tracer in dcLNs in the tested groups. The blood vessels were filled with Evans Blue (red). **b**–**d** Quantitative analysis of signal intensity from FITCD (a.u.) in ventral (**b**), dorsal (**c**) and in dcLNs (**d**) in the tested groups. 3-12-24 m means mouse age in months, a.u. means arbitrary units, n.s. means no significance; *n* = 10 in each group, the ANOVA test with the post hoc Duncan test

The results revealed age-related decline in brain drainage. So, the distribution of FITCD in the ventral and dorsal parts of the brain was 2.5-fold (*p* < 0.05) and 1.5-fold (*p* < 0.05) lesser in 12-month-old mice and 6.2-fold (*p* < 0.01) and 5.2 (*p* < 0.01) lesser in 24-month-old mice than in 3-month-old animals, respectively (Fig. 3a − c). A similar trend was observed in dcLNs. Indeed, the intensity signal from FITCD in dcLNs was 13-fold (*p* < 0.01) lesser in middle-aged and 17-fold (*p* < 0.01) lesser in old mice vs. young animals (Fig. 3a and d).

The findings demonstrate age-related increase in the Aβ brain levels. Indeed, the Aβ brain content was 2.5-fold (*p* < 0.001) higher in 12-month-old mice and 3.8-fold (*p* < 0.001) greater in 24-month-old mice compared to 3-month-old animals (5.52 ± 0.21 pg/mg vs. 2.21 ± 0.17 pg/mg, *p* < 0.001 between 12- and 3-month-old mice; 8.49 ± 2.45 pg/mg vs. 2.21 ± 0.17 pg/mg, *p* < 0.001 between 24- and 3-month-old mice, *n* = 10 in each group, the ANOVA test with the post hoc Duncan test) (Fig. 1h).

The 10-day course of PBM did not change the soluble Aβ content in the brains of young mice, but effectively reduced it in middle-aged mice to the level of young animals (2.23 ± 0.98 pg/mg vs. 2.21 ± 0.17 pg/mg, n.s. between 3-month-old with and without PBM; 2.32 ± 0.25 pg/mg vs. 5.52 ± 0.21 pg/mg, *p* < 0.001 between 12-month-old with and without PBM, *n* = 10 in each group, the ANOVA test with the post hoc Duncan test) (Fig. 1h). However, PBM did not affect the high soluble Aβ content in the brain of old mice (8.37 ± 2.04 pg/mg vs. 8.49 ± 2.45 pg/mg, n.s. between 24-month-old with and without PBM, *n* = 10 in each group, the ANOVA test with the post hoc Duncan test) (Fig. 1h).

Thus, there is a gradual decrease in brain drainage associated with the accumulation of Aβ in brain tissues that can be improved by PBM in middle-aged mice but not in old mice. The lack of effects of PBM on the brain soluble Aβ levels in young mice can be explained by its low content in the brain.

## Discussion

In this study, we clearly demonstrate age-related decline in cognitive function studying the formation of PCR in 3-, 12- and 24-month-old mice. Indeed, old mice performed the PCR test “light-lever-food” much longer than young and middle-aged animals, between which there was no difference. These data are consistent with other findings indicating memory deficit and cognitive dysfunction in old humans and animals [4, 5, 58–60]. PBM is considered as a method for improvement of cognitive function [6–12]. However, our results revealed that PBM effective modulated cognitive training exercises in young and middle-aged mice, but not in old animals. Indeed, 3- and 12-month-old mice receiving a course of PBM developed PCR “light-lever-food” significantly faster than intact animals, while old mice were insensitive to PBM. There were also no significant therapeutic effects in some old patients (85 years old) with idiopathic Parkinson’s disease and with mild- and moderate Alzheimer’s disease after PBM during 12 weeks [43, 44].

To investigate possible mechanisms of the lack of sensitivity to PBM in old mice, further our studies were focused on MLVs as an important target to PBM [13–18]. We found significant changes in the MLV morphology manifested in the form of lymphatic hyperplasia in 24-month-old mice. Ahn et al. also showed the overgrowth of the lymphatic endothelium of MLVs in old mice [20]. There is evidence that lymphatic hyperplasia is a compensatory response to the lymphatic valve dysfunction leading to a decrease in peristaltic lymphatic flow and brain drainage reduction [39–42].

To test age-related changes in brain drainage regulated by MLVs, we studied distribution of FITCD in brain tissues and lymphatic removal of tracer to dcLNs in mice of different ages. The results showed that spreading of FITCD on the dorsal and ventral surface of the brain as well as its accumulation in dcLNs were lesser in 24-month-old mice compared with 3- and 12-month-old animals.

The Aβ brain level is an indicator of effectiveness of brain drainage and the MLV functions [18, 19]. Therefore, we analyzed the Aβ content in the brains of mice of different ages for assessment of the stimulating PBM effects on brain drainage. The results revealed an age-related increase in the Aβ brain levels. Note that other studies have also found accumulation of Aβ in the brain of aged C57BL/6J mice [61]. A 10-day course of PBM effectively reduced the Aβ brain levels in middle-aged mice to the level of young mice, but had no effect on those in young or old mice. The lack of PBM effects on the Aβ brain levels in young mice may be due to its low levels, while in old mice it may be due to age-related changes in the MLVs and brain drainage, which provide Aβ clearance [19].

There is evidence suggesting correlation between extracellular deposition of Aβ throughout multiple cortical regions, aging and mild cognitive impairment [62]. Indeed, Aβ accumulation in the brain begins decades before the appearance of first clinical symptoms [63–65]. The increase in the Aβ brain levels triggers a pathological changes associated with synaptic dysfunction, neuroinflammation and neuronal loss leading to the irreversible cognitive and functional decline [63–65]. Therefore, deposition of Aβ in the brain is one of the earliest pathological markers associated with Alzheimer’s disease [66].

Despite the fact that Aβ deposition in the brain plays an important role in cognitive dysfunction and in the development of Alzheimer’s disease, it is still unclear whether it plays a key role in these pathophysiological mechanisms of memory and cognitive impairment in the aging brain and in patients with Alzheimer’s disease [67, 68]. Only 30% of cognitive deficits are associated with high Aβ deposits in the brain [69, 70]. Recent therapeutic strategies attempts at targeting Aβ plaques have failed to show the effectiveness in improvement of cognitive decline in patients with the late-stage of Alzheimer’s disease [71, 72]. However, recent studies confirm that Aβ accumulation in the brain correlates with a decline in cognitive function in old people and with moderate Alzheimer’s disease [62].

It has recently been proven that age-related changes in MLVs are the cause of Aβ accumulation in the brain, which correlates with a decline in cognitive function, as well as with the progression of Alzheimer’s disease [19]. Thus, the MLV dysfunction may be an aggravating factor in Alzheimer’s disease pathology and in age-associated cognitive decline [13–19, 73].

Our studies were performed only in male mice. This is because previous studies did not reveal sex differences in the functions and morphology of MLV as an important target in PBM [20]. Since we were answering the question of whether PBM could enhance cognitive function in old mice that lack reproductive function, sex differences in the effects of PBM on cognitive abilities and stimulation of brain drainage were not examined.

The main result of the study was the fact that the lack of sensitivity to PBM in old mice is combined with significant changes in the morphology of the MLS, reflecting a decrease in their drainage function.

The MLVs are located under the skull on the surface of the brain that makes MLVs accessible for PBM. The first studies in this direction were done using a 1267 nm laser, which stimulates direct ^1^O_2_ generation in living tissues [13, 14, 16, 35]. Pioneering studies have shown that the 1267 nm radiation stimulates the MLV functions. Indeed, PBM with 1267 nm laser stimulates lymphatic removal of blood from the mouse brain providing better recovery from intracranial hemorrhages [13]. The 1267 nm-activation of brain drainage contributes to increase in resistance to glioma progression [35] and microglia injury caused by diabetes mellitus [14]. 1267 nm-direct ^1^O_2_ generation also stimulates lymphatic clearance of Aβ, which improves cognitive function in mice with Alzheimer’s disease [16].

However, light sources emitting around 1270 nm are scarce and expensive, which makes them commercially unattractive [74]. In this regard, light irradiation 1065 nm is much more perspective due to the coincidence of this band with the light emitted by 1270 nm lasers. Despite the fact that for any biomedical purpose excitation at 1065 nm is equal to that at 1270 nm, there are two main fundamental and practical advantages for 1065 nm [74]. The 1065 nm irradiation has a tenfold reduction in water absorption as compared to 1270 nm, which allows 1065 nm to save more therapeutic energy. The 1065 nm absorption band coincides with the emission from commercially available lasers (~ 1064 nm) and light-emitting diodes (LEDs; ~ 1050 nm). Since LEDs are widely used in clinical practice for PBM and are recognized by the US Food and Drug Administration as safe technologies, as well as due to their commercially attractive price, they are the most promising for their implementation in clinical practice. Indeed, the first studies in this direction indicate the potential for using LED 1050 nm to effectively remove toxic Aβ from the brain in order to improve cognitive functions [15, 17, 18].

However, the question remains open: how to increase the effectiveness of PBM in old animals? Note that sleep significantly increases the therapeutic effects of PBM [15, 17, 18, 74]. This is largely due to the natural activation of brain drainage during deep sleep [75, 76]. Currently, there are no commercially available devices for PBM under sleep control [75]. A prototype of such a device has recently been proposed and tested in preclinical studies as a tool for both effective lymphatic removal of Aβ from the mouse brain and in improving cognitive function in mice with Alzheimer’s disease [18]. Sleep-based PBM technology has also proven effective in facilitating memory formation and new skills in healthy rodents [17]. This device is the world’s first technology that allows to control the MLV functions during sleep by photo-^1^O_2_ generation.

## Conclusion

Overall, this study clearly demonstrates that PBM is a promising method for modulation of cognitive functions and lymphatic clearance of Aβ in middle-aged mice. However, these effects of PBM are not evident in old mice, which are not sensitive to PBM. MLVs are an important target for PBM. We found morphological changes in the MLV network only in old mice in the form of lymphatic hyperplasia, which is considered a sign of decreased lymph flow. Based on this fact, we suggest that the preservation of the MLV functions is a determining factor in the effectiveness of the stimulatory effects of PBM on both Aβ clearance and cognitive functions. Recent studies have shown that MLV disruption blocks the effects of PBM on brain drainage and clearance of Aβ from brain tissue, which also indicates an important role of MLVs in the mechanisms of PBM-stimulating effects. Since deep sleep is a time of natural activation of brain drainage [76, 77] and sleep significantly enhances the stimulating drainage effects of PBM [15, 18], further studies on the effectiveness of PBM during sleep in old animals will shed light on improvement of therapeutic PBM effects at this age.

## Data Availability

The data that support the findings of this study are available on request from the corresponding author.
